# A Large Novel Deletion Downstream of *PAX6* Gene in a Chinese Family with Ocular Coloboma

**DOI:** 10.1371/journal.pone.0083073

**Published:** 2013-12-11

**Authors:** Hong Guo, Limeng Dai, Yanming Huang, Qiong Liao, Yun Bai

**Affiliations:** 1 Department of Medical Genetics, Third Military Medical University, Shapingba District, Chongqing, PR China; 2 Department of Ophthalmology, Xinqiao Hospital, Third Military Medical University, Shapingba District, Chongqing, PR China; Queen's University Belfast, United Kingdom

## Abstract

**Purpose:**

The paired box gene 6 (PAX6) is an essential transcription factor for eye formation. Genetic alterations in *PAX6* can lead to various ocular malformations including aniridia. The purpose of this study was to identify genetic defects as the underlying cause of familial ocular coloboma in a large Chinese family.

**Methods:**

After linkage analysis was carried out in this family, all exons of *PAX6* in the proband were sequenced by the Sanger sequencing technique. Then the genome of the proband was evaluated by a microarray-based comparative genomic hybridization (aCGH). Quantitative real-time PCR was applied to verify the abnormal aCGH findings.

**Results:**

All patients presented bilateral partial coloboma of iris, severe congenital nystagmus, hyperpresbyopia and congenital posterior polar cataracts. Two-point linkage analysis in the autosomal dominant family showed loss of heterozygosity at the D11S914 locus. There was no pathogenic mutation in the exons of *PAX6*. The aCGH analysis revealed a 681 kb heterozygous deletion on chromosome 11p13. Quantitative real-time PCR verified the deletion in the patients and further confirmed this deletion cosegregation with the ocular coloboma phenotype in the family.

**Conclusions:**

The 681 kb large deletion of chromosome 11p13 downstream of *PAX6* is the genetic cause of the familial ocular coloboma in this large Chinese family. aCGH should be applied if there is a negative result for the mutation detection of *PAX6* in patients with ocular coloboma.

## Introduction

Ocular Coloboma (OMIM 120200) is a congenital eye disorder characterized by partial absence of the iris and fundus coloboma. Most ocular coloboma are familial cases which are inherited as autosomal dominant, while the others without family history. Heterozygous mutations in the paired box gene 6 (*PAX6*, OMIM 607108), located on 11p13, were previously implicated in familial and sporadic ocular coloboma even aniridia (OMIM 106210) [1].

*PAX6* spans 22 kilobases and contains 14 exons encoding a protein with 422 amino acids. *PAX6* is a highly conserved transcriptional factor that controlled development of forebrain, pancreas and ocular tissues, including corneal epithelium, lens and retina [2]. To data, over 300 mutations of the *PAX6* gene caused different disease phenotypes through gain-of-function or loss-of-function, such as Aniridia (OMIM 106210), Cataract with late-onset corneal dystrophy (OMIM 106210), ocular coloboma (OMIM 120200), Coloboma of optic nerve (OMIM 120430), Morning glory disc anomaly (OMIM 120430), Foveal hyperplasia (OMIM 136520), Gillespie syndrome (OMIM 206700), Peters’ anomaly (OMIM 604229), Keratitis (OMIM 148190) and Optic nerve hypoplasia (OMIM 165550) [3]. Mutations or intragenic deletions of *PAX6* were the major causes of aniridia and iris coloboma, however, rare cases could be associated with large chromosomal deletions or rearrangements [4].

In the present study, we identified the genetic basis in a large Chinese family with ocular coloboma, using linkage analysis, microarray-based comparative genomic hybridization (aCGH) and quantitative real-time PCR.

## Materials and Methods

### Patients and genomic DNA extraction

**Figure 1:**
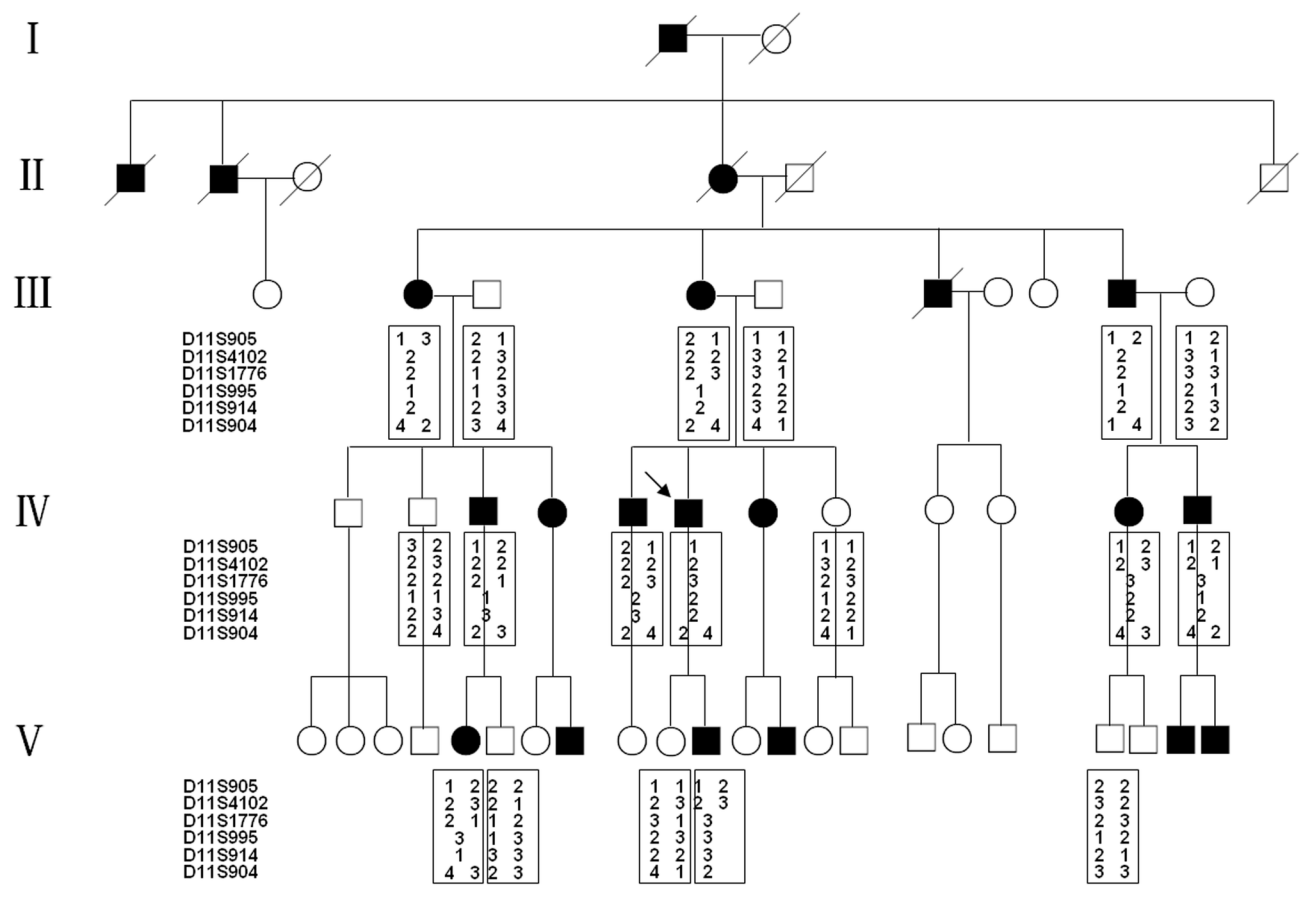
Pedigree and haplotype analysis of the family in this study. Squares and circles indicated males and females respectively. The symbols in black represent the affected members. The arrow indicates the proband. The square with a line indicated a deceased individual. Haplotype analysis showed loss of heterozygous segregation with D11S914 and D11S995. All markers were listed in descending order from the centromere on chromosome 11p.

The study was performed with the approval of the Ethics Committee of Third Military Medical University (Chongqing, China). The written informed consent obtained from the family members and the healthy controls to participate in this study. The participants of the family in this study were identified and enrolled at Xinqiao Hospital, Third Military Medical University, southwest of China. There were twenty-one affected individuals in this five-generation family (Figure 1), in which ten affected members and eight unaffected members participated in the study. Both the patients and the normal controls underwent ophthalmologic examination including bilateral naked eyes visual acuity and corrected visual acuity using E chart, slit-lamp microscopy inspection and intraocular pressure measurement. Some patients underwent electroretinography examination. Systemic evaluation was performed in the ten affected subjects in the study to exclude WAGR syndrome, iridocorneal endothelial syndromes, sclerocornea and Peter’s anomaly. The control group consisted of thirty healthy volunteers who showed no abnormalities on physical, neurological and ophthalmologic examinations. The venous blood samples were collected and drawn in Vacutainer tubes containing EDTA. Extraction of Genomic DNA was performed using Wizard Genomic DNA Purification Kit (Promega, USA) according to the protocol. The quantity and quality of DNA was determined by using NANODROP 1000 (Thermo, USA).

### Linkage and haplotype analysis

We carried out linkage analysis at the chromosome 11p. The family members were genotyped at 6 microsatellite marker loci that are distributed with an average of 5-cM intervals over the short arm of chromosome 11, D11S905, D11S4102, D11S1776, D11S995, D11S914, D11S904. Two-point LOD scores were calculated using the MLINK program of the FASTLINK package, assuming that the disease in the family was inherited in an autosomal dominant mode with complete penetrance (penetrance = 1.00), the disease-allele frequency was 0.0001 and allele frequencies were equal at all the marker loci.

### Sequencing of PAX6 gene

The software Primer3 was used to design the primers to amplify the whole 14 exons and the exon-intron boundaries of the *PAX6* gene. Conditions and the primer pairs for PCR are available upon request. PCR products were checked by 2% agarose gel electrophoresis and purified with purification kit (Tiangen, Beijing). Purified PCR products were directly sequenced in both forward and reverse directions by ABI 3130 genetic analyser (Applied Biosystems, Foster City, CA, USA) according to the manufacturer’s instructions. DNA sequences were analyzed using the vector NTI 11.0 software package.

### Microarray based comparative genomic hybridisation (array CGH)

Array comparative genomic hybridization (array CGH) was carried out in the proband by using the Roche NimbleGen Genome-Wide array CGH 3*720K containing over 720,000 copy-number probes. Genomic DNA samples were genotyped at the CapitalBio Corporation (Beijing, China) with the NimbleGen CGH array in accordance with the manufacturer’s protocols. Genotype calling, genotyping quality control, and CNV identification were performed with the Roche NimbleGen SignalMap software. The data could be accessed at Gene Expression Omnibus (GEO, No: GSE50577).

### Quantitative real-time PCR

Quantitative real-time PCR (qPCR) was performed to confirm the abnormal aCGH findings in the affected family members. Eight unaffected family members and thirty healthy controls were also evaluated by qPCR.Three primers sets for qPCR analysis were designed by using the Primer Express v2.0 software (Applied Biosystems, Foster City, California, USA), which contained primers targeted to *ELP4* gene in the deleted genomic region, primers within the *PAX6* gene and reference primers in the *ALB* gene. Both *ELP4* and *PAX6* were assayed as test genes compared with *ALB*. The qPCR was performed in a total volume of 20 ul in each tube containing 10 ul of SYBR Premix Ex Taq (TaKaRa), 5 ul of genomic DNA (50 ng), and 5 ul of primers (300 nM each) with three replicates per sample. Reaction run with the following conditions in ABI 7900HT (Applied Biosystems, Foster City, CA, USA): 95°C for 10 min and 40 cycles of 95°C 10 s/60°C 15 s/72°C 20 s. The relative copy number (RCN) was determined on the basis of the comparative ddCT method using a normal control DNA as the calibrator. Melt curve analysis was performed to confirm amplification specificity. The experiments were repeated three times. A cut-off RCN of 0.5 was used for deletion. T test was employed.

## Result

**Figure 2:**
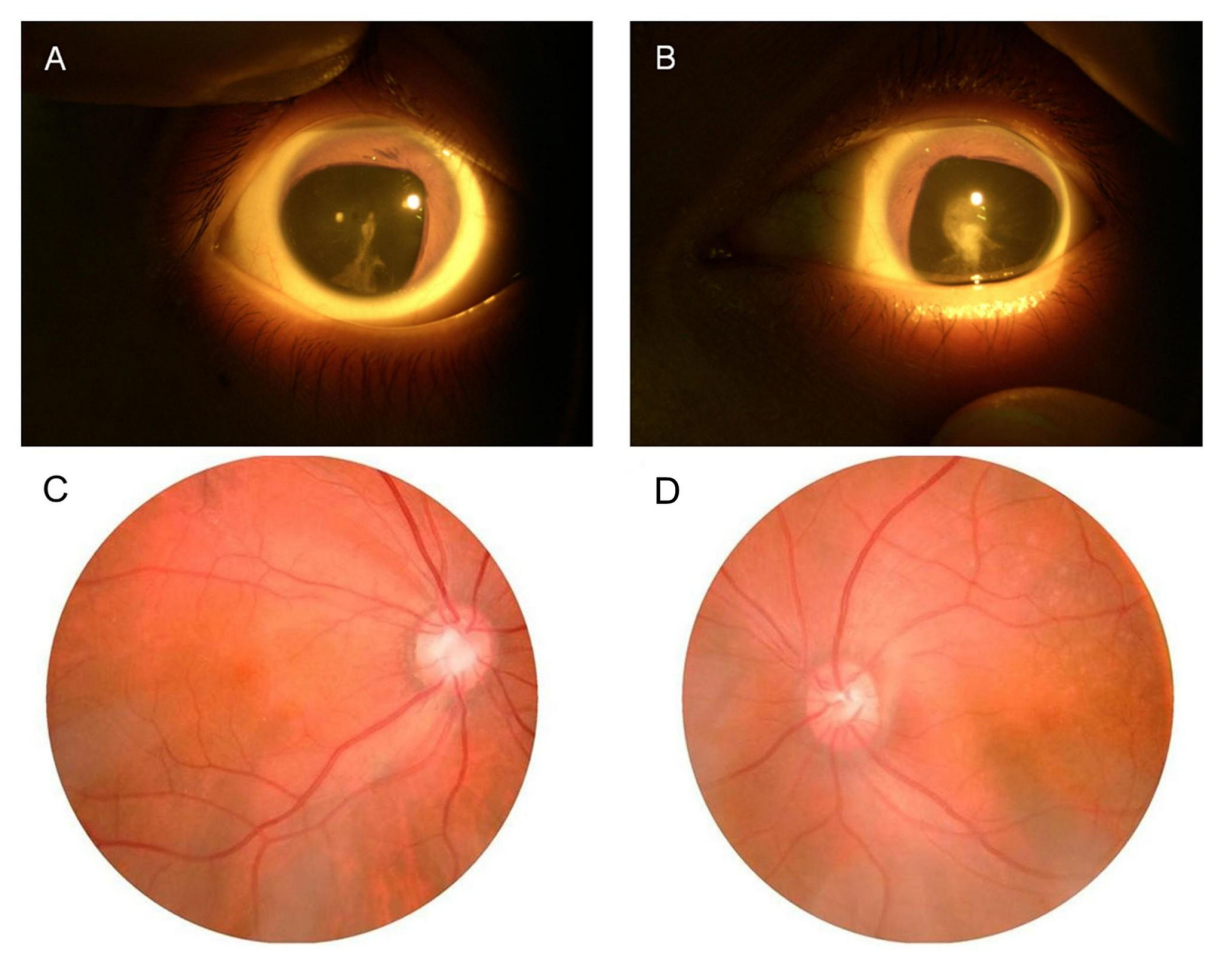
Ophthalmological findings in the proband. **A**-**B**: Photograph of anterior segment of the proband with partial coloboma of iris and congenital posterior polar cataract. **C**-**D**: No obvious abnormalities were found in the fundus images of the proband.

We identified a large family from southwest of China. Twenty-one family members had ocular coloboma in the family, in which sixteen patients were alive. Ten patients participated in the study, which presented bilateral partial coloboma of iris, hyperpresbyopia, severe congenital nystagmus and congenital posterior polar cataracts (Figure 2). No other obvious ophthalmic abnormalities were found. None of the patients developed secondary glaucoma or corneal changes. Neither mental retardation nor other general abnormalities was observed or documented in all patients in this family.

### Linkage analysis

In this family, the disease phenotype appeared in each generation, indicating that the disease of complete penetrance. Segregation analysis of the pedigree confirmed an autosomal dominant inheritance. Results of two-point linkage analysis were highly informative, which showed loss of heterozygosity at the D11S914 and D11S995 locus (Figure 1).

### Mutation analysis

By the direct sequencing of all 14 exons of *PAX6*, no intragenic point mutation or deletion was detected in the affected members in this family.

### The copy number variation analysis

**Figure 3:**
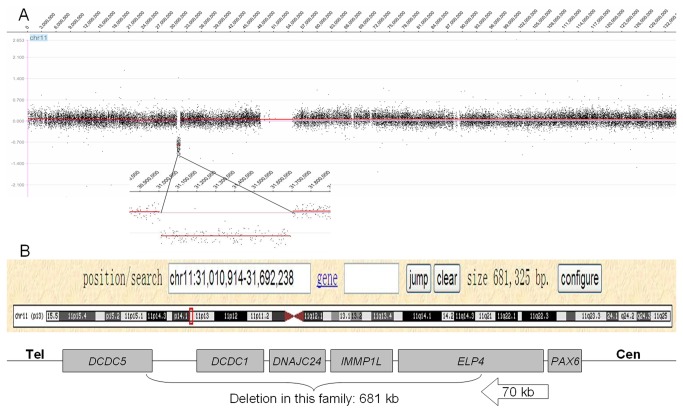
The 681 kb genomic deletion of chromosome 11p13. This deletion harbors five annotated genes, *DCDC5, DCDC1, DNAJC24, IMMP1L*, and *ELP4*.

Karyotype analyses were normal in the proband. However, the aCGH analysis detected a large deletion in the proband’s genome, which was a 681 kb heterozygous deletion of chromosome 11p13 (chr11: 31,010,914-31,692,238) with approximately a 70 kb distance from the 3′ end of PAX6 according to HG19 (NCBI 37, Feb 2009) (Figure 3). This deletion contained five annotated genes: doublecortin domain containing 5 (*DCDC5*), doublecortin domain containing 1 (*DCDC1*), DnaJ homolog subfamily C member 24 (*DNAJC24*), IMP1 inner mitochondrial membrane (*IMMP1L*), and elongation factor protein 4 (*ELP4*).

### Quantitative real-time PCR analysis

**Figure 4:**
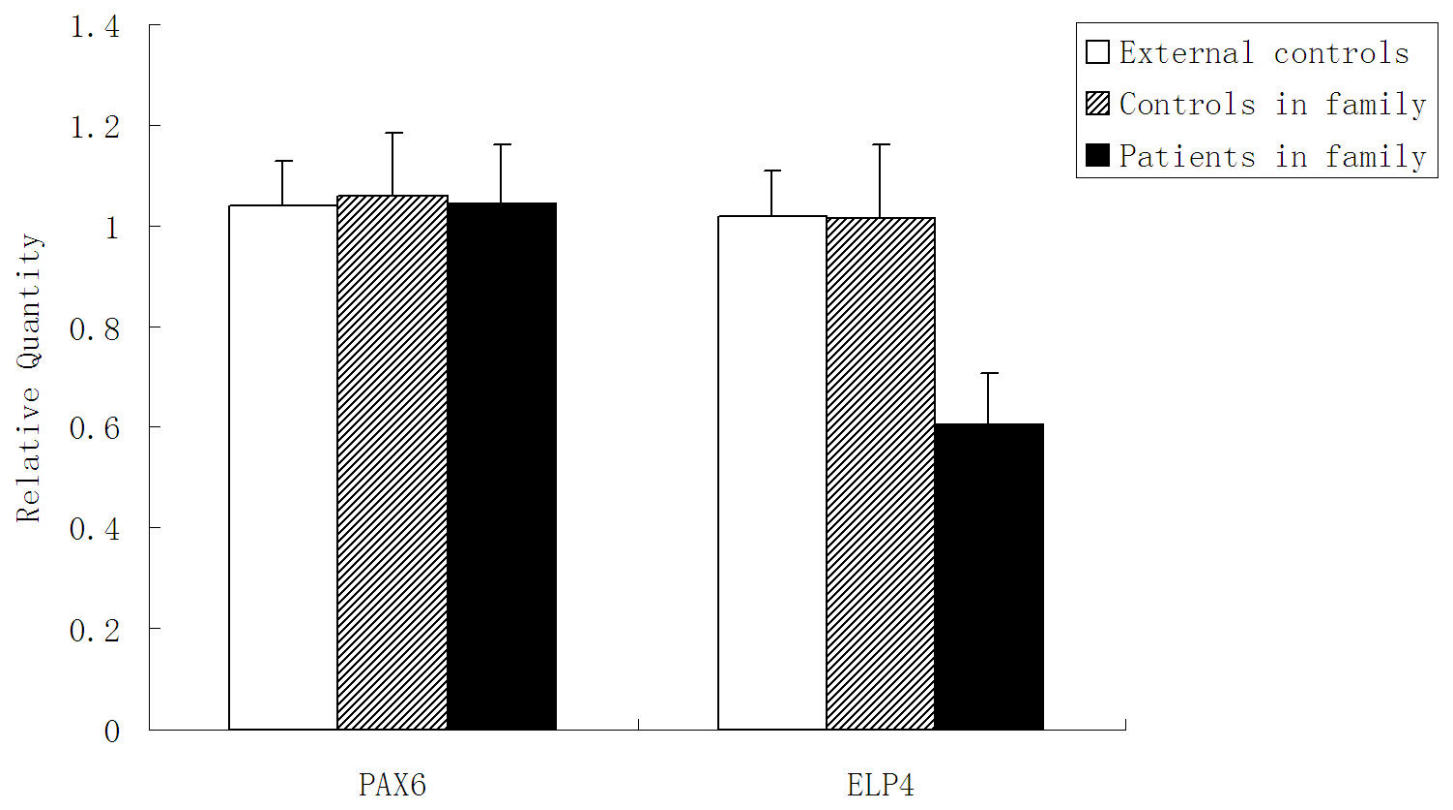
Confirming the genomic deletion downstream of *PAX6* gene by quantitative real-time PCR. Histogram indicated the relative quantity between *ELP4* and *PAX6* compared to *ALB* values in external controls, familial controls and familial patients.

Quantitative real-time PCR was conducted by using primers targeted to *ELP4* and *PAX6*. *ALB* was used to normalize *ELP4* and *PAX6* values for the detection of the relative copy number of the deletion region. Ten patients in the family, eight unaffected family members and thirty healthy external controls were examined. Two copies of *PAX6* were confirmed. We detected a half relative quantity (RQ) value for *ELP4* in affected individuals with respect to the normal controls (p<0.01), which verified the 681kb genomic deletion of chromosome 11p13 found by aCGH (Figure 4). This deletion also cosegregated with the disease phenotype of this family and suggested full penetrance of the disease.

## Discussion

Ocular coloboma and aniridia are allelic disorders related to heterozygous mutations in the paired box gene 6 (*PAX6*) [5]. Aniridia is characterized by partial or complete absence of the iris accompanied with other ocular abnormalities such as cataract and glaucoma. Ocular coloboma is associated with partial coloboma of iris with or without fundus coloboma and other ocular abnormalities. The two disorders occur predominantly through intragenic mutations, while copy number variations (CNVs) have also been reported.

In the present large Chinese family, we identified a novel heterozygous 681 kb deletion in the downstream flanking region of *PAX6*, which encompassed five known genes. The proximal breakpoint is approximately 70 kb from the 3′ end of *PAX6*. We postulated and verified that the deletion cosegregated and caused the ocular coloboma in this family. Submicroscopic CNVs in aniridia patients were previously described in several studies. Several studies in aniridia patients have proved that 3’ regulatory elements may be required for PAX6 transcription [6]. However, our findings firstly discovered deletion downstream of *PAX6* gene was associated with ocular coloboma.

Submicroscopic CNVs are common in the human genome [7]. Large deletions may involved in human disease either by disrupting coding regions or by affecting gene expression regulatory elements. Evolutionarily conserved enhancers that exhibit developmental stage-specific activity during eye development have been identified both 5’ and 3’ of the PAX6 gene [8]. Position effect is the underlying pathogenic mechanism of aniridia in these families. Moreover, analysis of the region with a breakpoint located 124 kb downstream of the PAX6 polyadenylation site using YAC transgenic mice, DNase I hypersensitivity mapping and reporter transgenic assays, proved the presence of several cis-regulatory elements, including a lens-specific element (SIMO element) and a retina-specific element. These elements are located within the introns of the *ELP4* gene and are *PAX6-*specific long-range control elements, called downstream regulatory region (DRR) [9,10]. Deletion of the lens-specific element in the DRR affects only expression in the lens, while deletion of the retina-specific element abolishes expression in the retina. Since retina specific enhancer within DRR must become active prior to other retina enhancers, the presence and proper functioning of the downstream regulatory region is essential for the PAX6 expression in this organ [11]. Our finding provides further evidence of the existence of the 3′ regulatory elements in the downstream region of *PAX6* controlling the expression of this gene.

The five genes found in the deleted area in this family are *DCDC5, DCDC1*, *DNAJC24*, *IMMP1L* and *ELP4*. Heterozygous or homozygous mutations of the genes have not been reported to be associated with any disease phenotypes. *DCDC5* and *DCDC1* is highly expressed in fetal brain. *IMMP1L* has peptidase activity and generates mature, active proteins in the mitochondrial intermembrane space. *DNAJC24* is one of several enzymes involved in the synthesis of diphthamide in translation elongation factor-2. *ELP4* is a ubiquitously expressed member of a complex of proteins that associates with histone acetyltransferases and RNA polymerase II to aid transcriptional elongation. Familial aniridia resulting from a deletion in the *ELP4* gene region was reported by D’Ellia firstly [4]. Then Davis et al [12] identified a 1.3 Mb deletion localized approximately 35 kb downstream of PAX6 in a patient with aniridia, autism and mental retardation. They stated that autism, in their case, may be caused by a combination of the PAX6 enhancer deletion and deletion of the other genes in the deleted region. Bayrakli et al [13], Cheng et al [14], Zhang et al [15] and Wawrocka et al [16] also identified 406.4 kb, 566 kb, 527 kb and 600 kb PAX6 3’ deletions in aniridia patients, which contained four genes *DCDC1*, *DNAJC24*, *IMMP1L* and *ELP4*. These patients had only aniridia and other ocular abnormalities, which is similar to the phenotype observed in most nonsense mutations patients. However, the major phenotype in our family is ocular coloboma. So we concluded that the disease phenotypes resulting from PAX6 3’ deletion might varied with the genes deleted in the region.

In this study, the ocular coloboma in the large Chinese family was caused by a large deletion in the PAX6 3’ region. We suggested that CNV should be investigated in the flanking regions of the PAX6 gene in congenital ocular anomalies. CGH technology has great advantages over standard karyotype methods and its use as a molecular diagnostic tool should be encouraged. Our results also demonstrated that linkage analysis should be carried out to confirm a genetic locus related to a phenotype in a family.

## References

[1] Gregory-Evans CY, Williams MJ, Halford S, Gregory-Evans K (2004) Ocular coloboma: a reassessment in the age of molecular neuroscience. J Med Genet 41(12): 881-891. doi:10.1136/jmg.2004.025494. PubMed: 15591273. 15591273 PMC1735648

[2] Georgala PA, Carr CB, Price DJ (2011) The role of Pax6 in forebrain development. Dev Neurobiol 71(8): 690-709. doi:10.1002/dneu.20895. PubMed: 21538923. 21538923

[3] Slavotinek AM (2011) Eye development genes and known syndromes. Mol Genet Metab 104(4): 448-456. doi:10.1016/j.ymgme.2011.09.029. PubMed: 22005280. 22005280 PMC3224152

[4] D'Elia AV, Pellizzari L, Fabbro D, Pianta A, Divizia MT et al. (2007) A deletion 3' to the PAX6 gene in familial aniridia cases. Mol Vis 13: 1245-1250. PubMed: 17679951. 17679951

[5] Kokotas H, Petersen MB (2010) Clinical and molecular aspects of aniridia. Clin Genet 77(5): 409-420. doi:10.1111/j.1399-0004.2010.01372.x. PubMed: 20132240. 20132240

[6] Lauderdale JD, Wilensky JS, Oliver ER, Walton DS, Glaser T (2000) 3' deletions cause aniridia by preventing PAX6 gene expression. Proc Natl Acad Sci U S A 97(25): 13755-13759. doi:10.1073/pnas.240398797. PubMed: 11087823. 11087823 PMC17648

[7] Hurles ME, Dermitzakis ET, Tyler-Smith C (2008) The functional impact of structural variation in humans. Trends Genet 24(5): 238-245. doi:10.1016/j.tig.2008.03.001. PubMed: 18378036. 18378036 PMC2869026

[8] Griffin C, Kleinjan DA, Doe B, van Heyningen V (2002) New 3' elements control Pax6 expression in the developing pretectum, neural retina and olfactory region. Mech Dev 112(1-2): 89-100. doi:10.1016/S0925-4773(01)00646-3. PubMed: 11850181. 11850181

[9] McBride DJ, Buckle A, van Heyningen V, Kleinjan DA (2011) DNaseI hypersensitivity and ultraconservation reveal novel, interdependent long-range enhancers at the complex Pax6 cis-regulatory region. PLOS ONE 6(12): e28616. doi:10.1371/journal.pone.0028616. PubMed: 22220192. 22220192 PMC3248410

[10] Kleinjan DA, Seawright A, Schedl A, Quinlan RA, Danes S et al. (2001) Aniridia-associated translocations, DNase hypersensitivity, sequence comparison and transgenic analysis redefine the functional domain of PAX6. Hum Mol Genet 10(19): 2049-2059. doi:10.1093/hmg/10.19.2049. PubMed: 11590122. 11590122

[11] Kleinjan DA, Seawright A, Mella S, Carr CB, Tyas DA et al. (2006) Long-range downstream enhancers are essential for Pax6 expression. Dev Biol 299(2): 563-581. doi:10.1016/j.ydbio.2006.08.060. PubMed: 17014839. 17014839 PMC2386664

[12] Davis LK, Meyer KJ, Rudd DS, Librant AL, Epping EA et al. (2008) Pax6 3' deletion results in aniridia, autism and mental retardation. Hum Genet 123(4): 371-378. doi:10.1007/s00439-008-0484-x. PubMed: 18322702. 18322702 PMC2719768

[13] Bayrakli F, Guney I, Bayri Y, Ercan-Sencicek AG, Ceyhan D et al. (2009) A novel heterozygous deletion within the 3' region of the PAX6 gene causing isolated aniridia in a large family group. J Clin Neurosci 16(12): 1610-1614. doi:10.1016/j.jocn.2009.03.022. PubMed: 19793656. 19793656

[14] Cheng F, Song W, Kang Y, Yu S, Yuan H (2011) A 556 kb deletion in the downstream region of the PAX6 gene causes familial aniridia and other eye anomalies in a Chinese family. Mol Vis 17: 448-455. PubMed: 21321669. 21321669 PMC3038207

[15] Zhang X, Zhang Q, Tong Y, Dai H, Zhao X et al. (2011) Large novel deletions detected in Chinese families with aniridia: correlation between genotype and phenotype. Mol Vis 17: 548-557. PubMed: 21364908. 21364908 PMC3044699

[16] Wawrocka A, Budny B, Debicki S, Jamsheer A, Sowinska A et al. (2012) PAX6 3' deletion in a family with aniridia. Ophthalmic Genet 33(1): 44-48. doi:10.3109/13816810.2011.615076. PubMed: 21985185. 21985185

